# Deep Metallic Surface Defect Detection: The New Benchmark and Detection Network

**DOI:** 10.3390/s20061562

**Published:** 2020-03-11

**Authors:** Xiaoming Lv, Fajie Duan, Jia-jia Jiang, Xiao Fu, Lin Gan

**Affiliations:** The State Key Lab of Precision Measuring Technology and Instruments, Tianjin University, Tianjin 300072, China; lvxiaoming1@gmail.com (X.L.); jiajiajiang@tju.edu.cn (J.-j.J.); fuxiao215@tju.edu.cn (X.F.); ganlin@tju.edu.cn (L.G.)

**Keywords:** surface defect detection, convolutional neural network, object detection

## Abstract

Metallic surface defect detection is an essential and necessary process to control the qualities of industrial products. However, due to the limited data scale and defect categories, existing defect datasets are generally unavailable for the deployment of the detection model. To address this problem, we contribute a new dataset called GC10-DET for large-scale metallic surface defect detection. The GC10-DET dataset has great challenges on defect categories, image number, and data scale. Besides, traditional detection approaches are poor in both efficiency and accuracy for the complex real-world environment. Thus, we also propose a novel end-to-end defect detection network (EDDN) based on the Single Shot MultiBox Detector. The EDDN model can deal with defects with different scales. Furthermore, a hard negative mining method is designed to alleviate the problem of data imbalance, while some data augmentation methods are adopted to enrich the training data for the expensive data collection problem. Finally, the extensive experiments on two datasets demonstrate that the proposed method is robust and can meet accuracy requirements for metallic defect detection.

## 1. Introduction

Surface defects have a greatly adverse effect on the quality of industrial products. Metallic defects detection has been exploited to satisfy predefined quality requirements for the industry. Therefore, metallic surface defect detection has attracted increasing interest in recent years and has achieved a positive improvement for the quality control in industrial applications [1]. However, metallic surface defect detection is easily influenced by many environmental factors such as illumination, light reflection, and metal material. These factors significantly increase the difficulty of surface defect detection.

**Figure 1:**
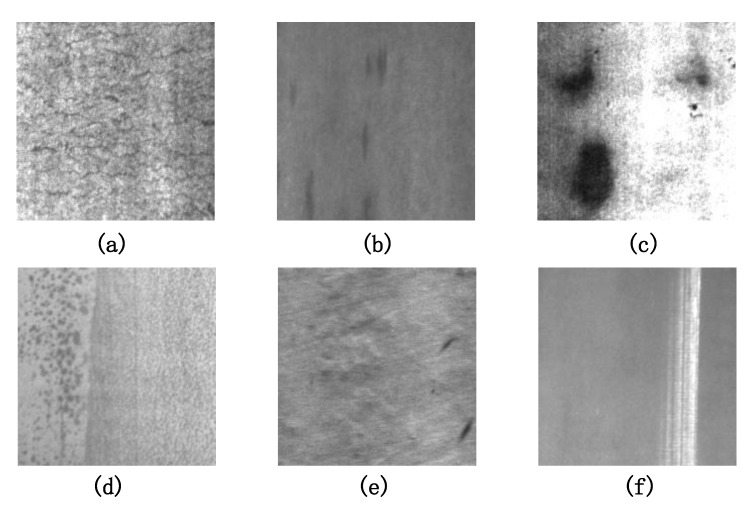
Some samples of Metallic surface defects. (**a**) Crazing. (**b**) Inclusion. (**c**) Patches. (**d**) Pitted surface. (**e**) Rolled in scale. (**f**) Scratches.

Several defects captured in the industry are shown in Figure 1. In the real-world environment, the defect types are varied and complex, including crazing, inclusion, patches, pitted surface, and scratches. However, most existing defect datasets are poor in data scale and defect richness, even limited to only a few categories. Specifically, the dataset size is generally limited to several hundred, which may lead to a detection model with weak robustness and generalization under complex industrial scenarios. To solve such a problem, it is necessary to introduce a new benchmark that is closer to realistic scenarios. Thus, we construct a new metallic surface defect dataset, named the “GC10-DET”.

In the real-world industrial environment, machine vision techniques are usually employed to detect metallic surface defects along the production line. Generally, these techniques refer to traditional image processing and deep learning that aim to analyze and detect defects collected in the manufactories. Although traditional image processing techniques have been successfully exploited to detect surface defects, deep learning-based approaches show great advantages in both surface defects detection and other industrial applications, such as the automotive industry [2], fruit classification [3], and object detection [4,5].

The main idea of traditional image processing techniques is to describe surface defects via well-designed hand-crafted features. The commonly used hand-crafted features contain LBP (local binary patterns), HOG (a histogram of the oriented gradient), GLCM (a gray level co-occurrence matrix), and other statistical features. For an input metallic surface image, the crucial point is to select suitable features to represent the defect information. According to the representation of the surface defects, a classifier is trained to recognize and classify the defects. These detection approaches have obtained a great improvement for various surface defect detections. However, traditional image processing methods cannot be directly deployed in reality, since they usually need complex threshold settings for defects recognition, which are sensitive to some environmental factors such as lighting conditions and background. If the environmental factors change, these threshold settings should be carefully adjusted again, otherwise, the algorithm is not applicable to the new environment due to lack of adaptability and robustness.

In this paper, we propose an end-to-end metallic surface defect network based on the Single Shot MultiBox Detector [6]. For each location on the feature map, the proposed model can separate the output space of the defect bounding boxes into a set of default boxes with different aspect ratios and multiple scales. For prediction, the proposed network generates confidence scores that denote the probabilities belonging to each object category for each default box. Besides, the proposed network can make suitable adjustments to search a better matching box. In addition, due to the significant imbalance between the positive and negative examples, we introduce a hard negative mining method to alleviate the problem of data imbalance. Furthermore, to solve the expensive data collection problem, we also adopt some data augmentation methods to enrich the training data. In summary, the main contributions of this paper are as follows:

- We contribute a new dataset named "GC10-DET" that includes 10 defect types collected in real industry situations.
- We propose a novel end-to-end defect detection and classification network based on the Single Shot MultiBox Detector combined with a hard negative mining method and data augmentation method.
- The extensive experiments on two datasets demonstrate the effectiveness of the proposed method and the superiority of our dataset.

## 2. Related Work

Numerous studies have been conducted for defect detection, yet, they have not been limited to metallic surfaces. These approaches can be mainly divided into two categories: the traditional methods and the deep learning methods, which are based on hand-crafted features or shallow learning techniques, respectively.

### 2.1. Traditional Method

Traditional methods mainly refer to traditional image processing techniques and shallow learning techniques (machine learning). Traditional image processing techniques extract hand-crafted features to describe and detect defects, which can be mainly divided into four categories: structural-based, threshold-based, spectral-based, and model-based methods [7]. In detail, the commonly used structural- based methods include skeleton-based [8], template match [9], edge-based [10], and morphological operations [11]. The threshold-based methods mainly contain the iterative optimal threshold [12], the Otsu method [13], contrast adjustment threshold method [14], and the Kittler method [15]. The spectral-based methods commonly consist of Fourier transform [16], wavelet transform [17], and Gabor transform [18], which are commonly used in image processing. Finally, model-based methods include the low-rank matrix model [19] and Gaussian mixture entropy model [20]. In general, shallow learning methods have two critical steps including feature extraction and classification. For an input surface image, hand-crafted methods are used to extract effective features for defect representation, then a special classifier is trained to judge whether the surface has defects. Local binary patterns (LBP) [21] and a histogram of oriented gradient (HOG) [22] are the most used features. There are lots of other features, such as co-occurrence matrix (GLCM) [23] and some grayscale statistical features [24,25]. However, the above detection methods cannot be directly deployed to the metallic surface, since traditional image processing techniques are very sensitive to illumination and background clutter. Multiple parameters need to be constantly adjusted for changed environmental factors; even the whole algorithm needs to be re-designed again. These approaches generally aim at only one specific environment, which is difficult to deploy in the more challenging real-world due to the lack of robustness and adaptability.

### 2.2. Deep Learning Method

Since the introduction of AlexNet [26], convolutional neural networks have been successfully deployed to detect surface defects. The authors in [27] outperformed classic computer vision approaches via combining hand-crafted features and support vector machines, which also demonstrate the superiority of deep learning in surface defect detection. However, this work was limited as they did not use ReLU and batch normalization in their network. Similarly, the authors in [28] proposed segmentation architecture for surface defect detection based on deep learning. In this work, ReLU was exploited as the activation function. In [29], the OverFeat network [30] was implemented to detect 5 different types of surface defects. The OverFeat network was trained on 1.2 million defect images from the ILSVRC2013 dataset including general objects. To compare deep networks with different amount of layers for surface detection, Weimer et al. [31] evaluated networks ranging from 5 layers to 11 layers. However, their method is inefficient since it extracted small patches and classified them respectively. Recently, Racki et al. [32] followed a two-stage segmentation network, in which several changes were conducted to increase the size of the receptive field. Racki et al. [32] and Weimer et al. [31] proposed to apply their networks to real-world samples rather than synthetic ones. However, the dataset only consists of a small number of defect images. Furthermore, there are other datasets reaching the hundreds or thousands. Lin et al. [33] proposed a LEDNet to exploit image-annotation and large batch sizes. This method must choose the network carefully since the number of training samples is an important factor that influences the performance of the detection system. The pre-trained models are often trained on ImageNet [34] and MS COCO [35] datasets.

## 3. Our Method

### 3.1. Overview of Our Industrial System

**Figure 2:**
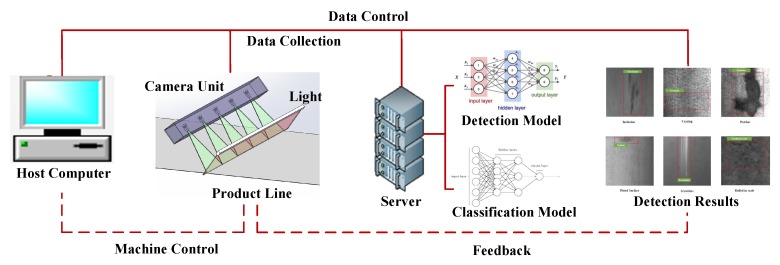
Overview of our industrial system. Our industrial system consists of host computers, production lines, servers, and detection results. The host computer is to control the operation of the entire system while the server is to deploy a defect detection model for the production line. Finally, detection results provide feedback for the production line.

Our industrial system consists of four major stages in a sequential manner: host computer, production line, server and detection results. The pipeline of the system architecture is shown in Figure 2. The host computer is the core of the system that controls the operation of the entire system. The production line is in the industry for defect image collection and production. We deploy our detection model on the server for quality estimation. Finally, we obtain detection results as feedback for the product line. For detail, the goal of the detection model is to detect and classify defects. The input original image is firstly transformed by several data augmentation methods. Secondly, these images are fed into the detection network for training, Our model can both detect and classify defects. Besides, a hard negative mining method is developed to speed up the convergence of the model. The entire system can be well deployed in the actual industrial environment.

### 3.2. Data Collection for Production Line

The data collection system consists of a set of linear array CCD cameras with a direct current (DC) light source to avoid the presence of stripes produced by an alternating current (AC). For some production lines, such as a hot-rolled strip production line, the running speed can achieve 10 m/s. Thus, the use of high-speed linear CCD cameras is able to improve the detection speed and the resolution of captured images. For a wide format steel plate, 4096 pixel line scan CCD cameras can be stitched to capture a complete image. The steel plate images are captured in this way and then we transmit these images to the server. The server exploits a large number of computing resources to detect the corresponding defects. Finally, results are output to the console for quality control.

To be rigorous, we introduce the brands, parameters and types of the related equipment for data collection as follows:

- **Camera**: The brand of camera is Teledyne while the camera model is DALSALA-CM-04K08A. The type of lens is ML-3528-43F of Moritex. The pixel size is 7.04 μm × 7.04 μm.
- **Server** The running memory is 32G with the GPU cards of NVIDIA RTX 2082ti.

### 3.3. Detection Model

**Figure 3:**
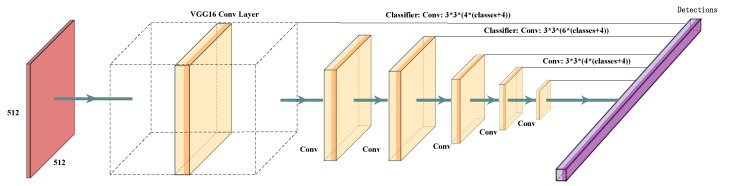
Overview of our detection network. Our model adds several Conv layers to the end of a base network, which predicts the offsets to default boxes of different scales and aspect ratios and their associated confidences.

As shown in Figure 3, the detection model is based on the Single Shot MultiBox Detector, which merely takes an input image and ground truth object boxes during the training process. In a convolutional fashion, the detection model adopts multi-scale feature maps to evaluate a set of boxes with different aspect ratios at each position. For each box, the network predicts both the offsets and the confidences for each category. During training, these boxes are matched with the ground truth boxes. The loss is a weighted sum between Smooth L1 and Softmax Loss. The base of the detection model is a feed-forward convolutional neural network, consisting of two major modules: VGG16 model and a non-maximum suppression procedure to output the final detection results. We then add extra architectures into the network including multi-scale feature maps and predictors for detection.

#### 3.3.1. Multi-Scale Feature Maps

Several convolutional layers are added to the end of the base VGG16 network. The goal is to progressively decrease the feature size and detect at multiple scales.

#### 3.3.2. Predictors for Detection

Each convolutional layer in our network can produce a fixed set of predicted parameters using a set of convolutional filters. The basic element to predict detections is a 3×3×c convolution kernel, where *c* is the number of the channels. The convolution kernel is used to produce the confidence for categories of each box. For each location of the feature map, it is applied to output the value.

### 3.4. Defect Default Boxes

At the top network, a set of defect default bounding boxes are matched with each feature map cell for multi-scale feature maps. The feature map is produced by convolutional filters to associate with defect boxes; thus, each box position is relative to both fixed and corresponding cells. In each feature map cell, we predict the offsets for the defect box and confidence scores that indicate the probabilities belonging to each category. Besides, at a given location, we calculate *c* class scores and the 4 offsets corresponding to the original default box shape. Thus, total (c+4)k convolutional filters are needed to produce the feature maps, where *k* is the number of default boxes. Therefore, an m×n feature map has (c+4)kmn outputs.

### 3.5. Loss Function

The training objective is inspired by the MultiBox objective [6] to handle multiple object classes. We use $x_{ij}^{p}$ to indicate if *i*-th default box matches with the *j*-th ground truth box of category *p*. If matched, let $x_{ij}^{p}=1$, otherwise, let $x_{ij}^{p}=0$. Thus, we obtain $\sum_{i}x_{ij}^{p}\geq 1$. The overall loss function is a combination of the localization loss and the confidence loss via weighted sum, which can be written as:

(1) $$L\left(x,c,p,g\right)=\frac{1}{N}\left(L_{loc}\left(x,c\right)+\alpha L_{conf}\left(x,p,g\right)\right)$$

where *N* is the number of the matched default boxes, *c* is the center of the box, *p* is the predicted box and *g* is the ground truth. Besides, if there are no matched boxes (N=0), the loss is set as 0. Then, we regress to offsets for the center ($c_{x},c_{y}$), width (*w*), and height (*h*) of the default box (*d*). Thus, the localization loss is written as:

(2) $$\begin{array}{cc} \; & L_{loc}\left(x,p,g\right)=\sum_{i}\sum_{m}x_{ij}^{k}{\mathrm{smooth}}_{L1}\left(p_{i}^{m}-{\hat{g}}_{j}^{m}\right)\\ \; & {\hat{g}}_{j}^{c_{x}}=\left(g_{j}^{c_{x}}-d_{i}^{c_{x}}\right)/d_{i}^{w}\;{\hat{g}}_{j}^{cy}=\left(g_{j}^{c_{y}}-d_{i}^{c_{y}}\right)/d_{i}^{h}\\ \; & {\hat{g}}_{j}^{w}=\log\left(\frac{g_{j}^{w}}{d_{i}^{w}}\right)\;{\hat{g}}_{j}^{h}=\log\left(\frac{g_{j}^{h}}{d_{i}^{h}}\right) \end{array}$$

where *i* is the indicator of the positive samples and $m\in \{c_{x},c_{y},w,h\}$. The confidence loss is a softmax loss for multiple classes and their confidence (c). It can be written as:

(3) $$L_{\mathrm{conf}}\left(x,c\right)=-\sum_{i\in pos}^{N}x_{ij}^{p}\log\left({\hat{c}}_{i}^{p}\right)-\sum_{i\in neg}\log\left({\hat{c}}_{i}^{0}\right)$$

where

(4) $${\hat{c}}_{i}^{p}=\frac{\exp\left(c_{i}^{p}\right)}{\sum_{p}\exp\left(c_{i}^{p}\right)}$$

and the weight term α is set as 1 via cross validation.

### 3.6. Matching Strategy

During the training process, the corresponding ground truth is to be selected from default boxes for the loss computation. These selected ground truth boxes vary over different aspect ratios and scales. Inspired by [36], we match default boxes to any ground truths according to a Jaccard overlap that is higher than a threshold. This operation allows the network to output high prediction scores for multiple overlapping boxes rather than selecting only the one with maximum overlap.

### 3.7. Hard Negative Mining

It is obvious that most of the default boxes are negatives after matching, especially when the number of default boxes becomes large. This would introduce a significant bias because of the imbalance between the positive and negative training samples. To solve this problem, we exploit their confidence loss to choose the highest confidence default boxes so that the ratio between the negatives and positives is limited (at most 3:1). This can lead to a more stable and faster training.

### 3.8. Data Augmentation

In order to obtain a robust model for various shapes and size of the object, we make a data augmentation for each training image like [ssd] as follows: (1) Use the entire original image. (2) Select a patch so that the minimum Jaccard overlap with the objects is 0.1, 0.3, 0.5, 0.7, or 0.9. (3) Randomly select a patch. (4) The size of each selected patch is [0.1,1] of the original size. The aspect ratio is between 1/2 and 2.

## 4. Experiments

In this section, we conduct a series of experiments to evaluate the proposed method using real defect images of a metallic surface. First, we provide a brief introduction of the used datasets and experimental settings. Then, the experimental results are presented in both visual and quantitative analyses. Finally, we conclude the whole work and present future work.

### 4.1. Datasets

#### 4.1.1. Description of NEU-DET

NEU-DET [21] is the Northeastern University (NEU) surface defect dataset that includes six types of surface defects, i.e., rolled-in scale (Rs), patches (P), crazing (Cr), pitted surface (Ps), inclusion (In) and scratches (Sc). The collected defects are on the surface of the hot-rolled steel strip. The dataset includes 1800 gray-scale images, i.e., 300 samples in each class of surface defects. The detailed defects are as follows:

- **Inclusion:** Inclusion is a typical defect of metal surface defects. Some inclusions are loose and easy to fall off, some pressed into the plate.
- **Crazing:** Crazing is the phenomenon that produces some cracks on the surface of a material.
- **Patches:** A part of metal marked out from the rest by a particular characteristic.
- **Pitted surface:** Pitting is a form of corrosion that focuses on a very small range of metal surfaces and penetrates into the metal interior. Pitting is generally small in diameter but deep in depth.
- **Scratches:** A scratch is a mark of abrasion on a surface.
- **Rolled in scale:** A rolled-in scale defect occurs when the mill scale is rolled into the metal during the rolling process.

#### 4.1.2. Description of GC10-DET

The GC10-DET dataset is available on the github (**Website:** https://github.com/lvxiaoming2019/GC10-DET-Metallic-Surface-Defect-Datasets). GC10-DET is the surface defect dataset collected in a real industry. It contains ten types of surface defects, i.e., punching (Pu), weld line (Wl), crescent gap (Cg), water spot (Ws), oil spot (Os), silk spot (Ss), inclusion (In), rolled pit (Rp), crease (Cr), waist folding (Wf). The collected defects are on the surface of the steel sheet. The dataset includes 3570 gray-scale images. Table 1 shows the comparison of NEU-DET and GC10-DET dataset. The detailed defects are as follows:

- **Punching:** In the production line of the strip, the steel strip needs to be punched according to the product specifications; mechanical failure may lead to unwanted punching, resulting in punching defects.
- **Welding line:** When the strip is changed, it is necessary to weld the two coils of the strip, and the weld line is produced. Strictly speaking, this is not a defect, but it needs to be automatically detected and tracked to be circumvented in subsequent cuts.
- **Crescent gap:** In the production of steel strip, cutting sometimes results in defects, just like half a circle.
- **Water spot:** A water spot is produced by drying in production. Under different products and processes, the requirements for this defect are different. However, because the water spots are generally with low contrast, and are similar to other defects such as oil spots, they are usually detected by mistake.
- **Oil spot:** An oil spot is usually caused by the contamination of mechanical lubricant, which will affect the appearance of the product.
- **Silk spot:** A local or continuous wave-like plaque on a strip surface that may appear on the upper and lower surfaces, and the density is uneven in the whole strip length direction. Generally, the main reason lies in the uneven temperature of the roller and uneven pressure.
- **Inclusion:** Inclusion is a typical defect of metal surface defects, usually showing small spots, fish scale shape, strip shape, block irregular distribution in the strip of the upper and lower surface (global or local), and is often accompanied by rough pockmarked surfaces. Some inclusions are loose and easy to fall off and some are pressed into the plate.
- **Rolled pit:** Rolled pits are periodic bulges or pits on the surface of a steel plate that are punctate, flaky, or strip-like. They are distributed throughout the strip length or section, mainly caused by work roll or tension roll damage.
- **Crease:** A crease is a vertical transverse fold, with regular or irregular spacing across the strip, or at the edge of the strip. The main reason is the local yield along the moving direction of the strip in the uncoiling process.
- **Waist folding:** There are obvious folds in the defect parts, a little more popular, a little like wrinkles, indicating that the local deformation of the defect is too large. The reason is due to low-carbon.

### 4.2. Performance Evaluation

We adopt Recall, Average Precision (AP), and mean Average Precision (mAP) for performance evaluation. Recall represents the ratio of correctly detected images and all testing images for each defect category. AP represents the average detected precision for each defect category; mAP is the mean of average detected precision for all defect categories.

### 4.3. Comparison Methods and Parameter Tuning

We compared the proposed model with several state-of-the-art methods, including: SSD [6], Faster-RCNN [37], YOLO-V2 [38], and YOLO-V3 [39]. Besides, we also compared proposed model with several traditional methods, including: LBP [21] and HOG [22]. The classifiers are the nearest neighbor classifier (NNC) and the support vector machine (SVM).

To be rigorous, we introduce the best parameter tuning process in this section. The above-mentioned deep methods adopt a pre-trained model on the ImageNet, which can be helpful to extract basic image features including edge, texture and so on. Therefore, the SSD method utilizes VGG16 as the pre-trained model, YOLO-V2 uses Darknet19 model, YOLO-V3 uses Darknet53 model, and Faster R-CNN adopts Resnet50 model. We claim parameter tuning for them respectively, as follows:

- **Learning Rate:** In the classical back propagation algorithm, the learning rate is determined by training experience. The larger training rate denotes the larger weight updating, which can accelerate the convergence of the model, but if the learning rate is too large, it may cause the oscillation of the training. Besides, a slower learning rate may lead to a slow convergence of the training process. Thus, we adjust as follows: (1) A large learning rate is used to initialize the model, and the learning rate decreases as training iterations increase. (2) Initial learning rate is set from 0.1 to 0.00001, and the best one is selected through experiments. Thus, we obtain the best learning rate as follows: SSD (0.0005), Faster-RCNN (0.01), YOLO-V2 (0.0005), and YOLO-v3 (0.0005).
- **Weight decay:** Weight decay is used to alleviate overfitting. In the loss function, the weight decay is a coefficient of the regular term. Thus, the setting of weight decay depends on the loss function. According to the loss function and experiments, the final settings of weight decay are follows: SSD (0.00005), Faster-RCNN (0.0001), YOLO-V2 (0.0001), and YOLO-v3 (0.00005).
- **Momentum:** Momentum is a method to retrieve the updating direction and speed up convergence of the model. This value is fixed for the SGD method according to the existing experiments. Thus, all of the methods have a set momentum of 0.9.

### 4.4. Accuracy Comparisons with Deep Methods

#### 4.4.1. NEU-DET

**Figure 4:**
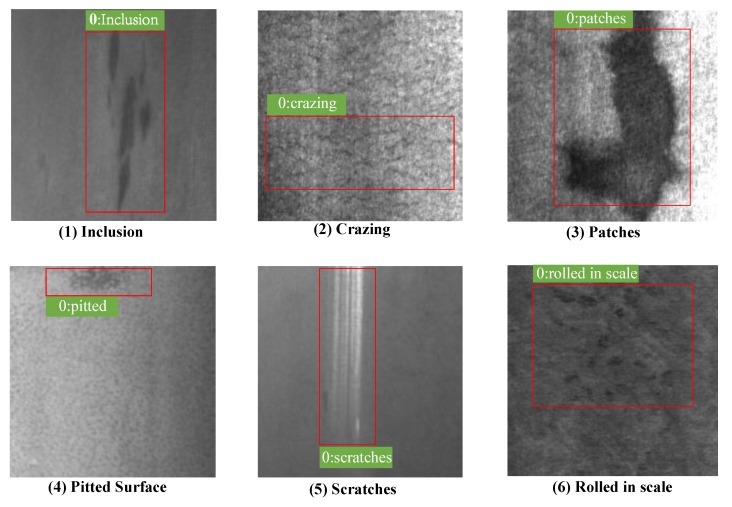
Detection results on NEU-DET dataset. In sequence, the pictures are: (**1**) Inclusion, (**2**) Crazing, (**3**) Patches, (**4**) Pitted surface, (**5**) Scratches, and (**6**) Rolled in scale.

Table 2 shows the detailed comparison results of Recall on the NEU-DET dataset. Some detection results of NEU-DET are shown in Figure 4. The proposed method can obtain the best results on the defects of Cr, In, Pa, Ps, and Rs, while the SSD300 is slightly higher than proposed method on Sc (0.990 vs. 0.981). Table 3 shows the detailed comparison results of AP and mAP on the NEU-DET dataset. The proposed method can obtain the best results on the defects of Cr, Pa, Ps, Rs, and Sc, while the SSD300 is slightly higher than the proposed method on In (0.796 vs. 0.763) and Rs (0.621 vs. 0.581).

As shown in Table 2 and Table 3, the YOLO methods are difficult to distinguish the six types of defects. The reason may be because the defects on the surface generally are small scale, which cannot be well solved by YOLO-V2 and YOLO-V3 with fixed scale detection. However, the proposed method adopts multi-scale cells to better distinguish multi-scale defects and the mAP can reach 0.724. While Faster-RCNN exploits anchor boxes to overcome this problem, it is still lower than the proposed method.

#### 4.4.2. GC10-DET

**Figure 5:**
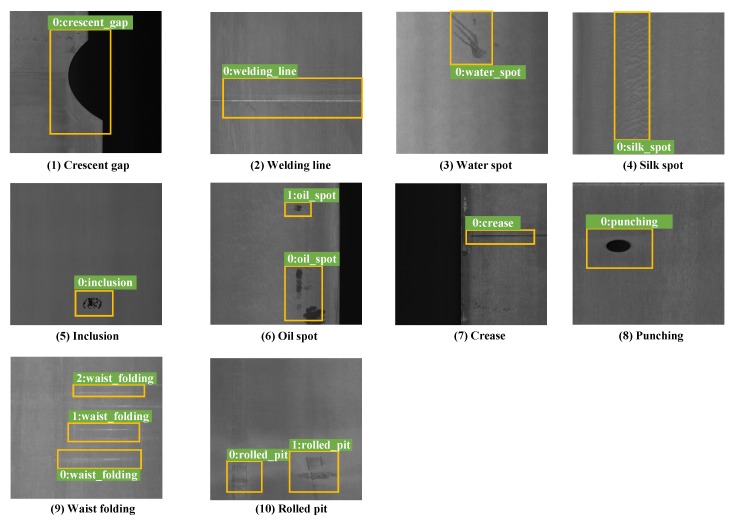
Detection results on the GC10-DET dataset. In sequence, the pictures are: (**1**) Crescent gap, (**2**) Welding line, (**3**) Water spot, (**4**) Silk spot, (**5**) Waist folding, (**6**) Inclusion, (**7**) Oil spot, (**8**) Crease, (**9**) Punching, and (**10**) Rolled pit.

Table 4 shows the detailed comparison results of Recall on the GC10-DET dataset. Some detection results of GC10-DET are shown in Figure 5. The proposed method can obtain the best results on the defects of Pu, Wl, Cg, Ws, Os, Ss, In and Wf, while the SSD300 is slightly higher than proposed method on Rp (0.667 vs. 0.333) and Faster-RCNN is higher than proposed on Cr (1 vs. 0.857).

Table 5 shows the detailed comparison results of AP and mAP on the GC10-DET dataset. The proposed method can obtain the best results on the defects of Pu, In, and Rp, while the SSD300 is slightly higher than proposed on Wl (0.974 vs. 0.885), Ss, (0.689 vs. 0.650) and Wf (1 vs. 0.919). Faster-RCNN is higher than proposed on Cg (0.872 vs. 0.848), Ws (0.599 vs. 0.558), Os (0.653 vs. 0.622), and Cr (0.736 vs. 0.521). Besides, the proposed method shows the best mAP accuracy.

As shown in Table 2 and Table 3, the YOLO methods are difficult to distinguish between the six types of defects. The reason may be because the defects on the surface generally are small scale, which cannot be well solved by YOLO-V2 and YOLO-V3 with fixed scale detection. However, proposed method adopts multi-scale cells to better distinguish multi-scale defects, and the mAP can reach 0.724. While Faster-RCNN exploits anchor boxes to overcome this problem, it is still lower than proposed method.

### 4.5. Accuracy Comparisons with Traditional Methods

Table 6 shows the detailed comparison results of precision on the NEU-DET dataset. The proposed method can obtain the best results on the defects of Cr, In, Pa, Ps, Rs, and Sc, while traditional methods had the worse results. It is noticed that different hand-crafted features provided different results, because the representation of one hand-crafted feature is limited. Although we try our best to assign the parameters of the traditional methods such as threshold, they still performed worse than the proposed method, which uses deep convolutional network.

### 4.6. Computational Time Comparisons

As shown in Table 7, the proposed method can work with a relatively fast speed. To process one image of NEU-DET, the proposed method performed a similar computational time to SSD, i.e., 27 ms vs. 29 ms, while the result is 6 s vs. 7 s for the whole testing set. On the GC10-DET, to process one image, the proposed method performed a second computational speed, i.e., 33 ms vs. 29 ms (SSD), while results for whole testing set came third with 8 s vs. 4.49 s (YOLO-V2). Although the computational time of YOLO-V2 may be slightly smaller than the proposed method, the accuracy of the proposed method is higher. In addition, as shown in Table 8, the traditional methods generally cannot meet the requirements in real-time.

## 5. Conclusions

In this paper, we contribute a new dataset called GC10-DET for metallic surface defect detection. The GC10-DET dataset has various challenges regarding defect types, defect images, and dataset scales. Besides, we propose an end-to-end defect detection and classification network based on the Single Shot MultiBox Detector. To solve the significant imbalance between the positive and negative examples, we present a hard negative mining method to effectively train our network. Furthermore, to enrich the training data, we also introduce some data augmentation methods into our training. Finally, extensive experiments demonstrate that the proposed method is robust for metallic defect detection. 

## Figures and Tables

**Table 1:** Comparison of NEU-DET and GC10-DET dataset.

Dataset	Scale	Type Number	Defect Types
NEU-DET	1800	6	rolled-in scale, patches, crazing, pitted surface, inclusion, scratches
GC10-DET	3570	10	punching, weld line, crescent gap, water spot, oil spot, silk spot, inclusion, rolled pit, crease, waist folding

**Table 2:** Comparison of Recall on NEU-DET dataset. The proposed method performs the highest Recall values for five defect categories. The bold helps to emphasize the highest data.

Types	Recall				
	SSD	Faster-RCNN	YOLO-V2	YOLO-V3	Proposed Method
Cr	0.965	0.874	0.552	0.692	**0.965**
In	0.974	0.923	0.811	0.755	**0.974**
Pa	0.935	0.981	0.910	0.923	**0.987**
Ps	0.971	0.943	0.791	0.561	**1.000**
Rs	0.932	0.881	0.500	0.602	**0.966**
Sc	**0.990**	0.971	0.884	0.816	0.981

**Table 3:** Comparison of Average Precision (AP) on NEU-DET dataset. The proposed method performs the highest AP values for four defect categories. The proposed method also provides the highest mAP value. The bold helps to emphasize the highest data.

AP	Recall				
	SSD	Faster-RCNN	YOLO-V2	YOLO-V3	Proposed Method
Cr	0.411	0.374	0.211	0.221	**0.417**
In	**0.796**	0.794	0.592	0.580	0.763
Pa	0.839	0.853	0.774	0.772	**0.863**
Ps	0.839	0.815	0.454	0.239	**0.851**
Rs	**0.621**	0.545	0.246	0.335	0.581
Sc	0.836	0.882	0.739	0.570	**0.856**
**mAP**	**0.724**	0.711	0.503	0.453	**0.724**

**Table 4:** Comparison of Recall on GC10-DET dataset. The proposed method performs the highest Recall values for seven defect categories. The bold helps to emphasize the highest data.

Types	Recall				
	SSD	Faster-RCNN	YOLO-V2	YOLO-V3	Proposed Method
Pu	0.964	0.964	0.857	0.964	**0.965**
Wl	1.000	0.623	0.869	0.869	**0.967**
Cg	0.968	0.968	0.936	0.871	**0.969**
Ws	0.696	0.696	0.674	0.609	**0.739**
Os	0.848	0.761	0.630	0.565	**0.891**
Ss	0.956	0.708	0.694	0.542	**0.988**
In	0.578	0.551	0.444	0.311	**0.667**
Rp	**0.667**	0.333	0.333	0.333	0.333
Cr	0.571	**1.000**	0.429	0.429	0.857
Wf	**1.000**	0.800	0.900	0.700	**1.000**

**Table 5:** Comparison of Average Precision (AP) on GC10-DET dataset. The proposed method performs the highest AP values for three defect categories. The proposed method also provides the highest mAP value. The bold helps to emphasize the highest data.

Types	AP				
	SSD	Faster-RCNN	YOLO-V2	YOLO-V3	Proposed Method
Pu	0.860	0.899	0.725	0.836	**0.900**
Wl	**0.974**	0.554	0.328	0.241	0.885
Cg	0.861	**0.872**	0.819	0.752	0.848
Ws	0.552	**0.599**	0.476	0.495	0.558
Os	0.612	**0.653**	0.403	0.329	0.622
Ss	**0.689**	0.579	0.473	0.325	0.650
In	0.168	0.194	0.096	0.036	**0.256**
Rp	0.105	0.364	0.018	0.036	**0.364**
Cr	0.527	**0.736**	0.212	0.429	0.521
Wf	**1.000**	0.818	0.614	0.400	0.919
mAP	0.635	0.627	0.433	0.388	**0.651**

**Table 6:** Comparison results with traditional methods on NEU-DET dataset. The bold helps to emphasize the highest data.

Types	AP				
	LBP + NNC	LBP + SVM	HOG + NNC	HOG + SVM	Proposed Method
Cr	0.321	0.335	0.400	0.412	**0.417**
In	0.412	0.378	0.576	0.580	**0.763**
Pa	0.538	0.601	0.612	0.630	**0.863**
Ps	0.446	0.515	0.438	0.328	**0.851**
Rs	0.237	0.330	0.358	0.330	**0.581**
Sc	0.326	0.432	0.460	0.500	**0.856**
**mAP**	0.380	0.432	0.474	0.463	**0.724**

**Table 7:** Comparison of Computational Time on Two datasets for deep methods.

Dataset	AP				
	LBP + NNC	LBP + SVM	HOG + NNC	HOG + SVM	Proposed Method
NEU-DET	379.65 ms	378.56 ms	465.32 ms	453.61 ms	27 ms
GC10-DET	399.01 ms	391.08 ms	495.26 ms	492.75 ms	33 ms

**Table 8:** Comparison of Computational Time on Two datasets for traditional methods.

Dataset	Type	Method				
		SSD	Faster-RCNN	YOLO-V2	YOLO-V3	Proposed Method
NEU-DET	single image	29 ms	37 ms	7.91 ms	15.75 ms	27 ms
	testing set	7 s	7 s	4.03 s	8.46 s	6 s
GC10-DET	single image	29 ms	43 ms	78.01 ms	86.80 ms	33 ms
	testing set	5 s	11 s	4.49 s	8.67 s	8 s

## References

[1] Kim S., Kim W., Noh Y.K., Park F.C. Transfer Learning for Automated Optical Inspection. Proceedings of the 2017 International Joint Conference on Neural Networks (IJCNN).

[2] Luckow A., Cook M., Ashcraft N., Weill E., Djerekarov E., Vorster B. Deep Learning in the Automotive Industry: Applications and Tools. Proceedings of the 2016 IEEE International Conference on Big Data (Big Data).

[3] Hossain M.S., Al-Hammadi M., Muhammad G. (2018). Automatic fruit classification using deep learning for industrial applications. IEEE Trans. Ind. Inf..

[4] Yang Z., Mahajan D., Ghadiyaram D., Nevatia R., Ramanathan V. Activity Driven Weakly Supervised Object Detection. Proceedings of the IEEE Conference on Computer Vision and Pattern Recognition.

[5] Benhimane S., Najafi H., Grundmann M., Genc Y., Navab N., Malis E. Real-Time Object Detection and Tracking for Industrial Applications. Proceedings of the Third International Conference on Computer Vision Theory and Applications.

[6] Liu W., Anguelov D., Erhan D., Szegedy C., Reed S., Fu C.Y., Berg A.C. Ssd: Single Shot Multibox Detector. Proceedings of the European conference on computer vision.

[7] Ren R., Hung T., Tan K.C. (2017). A generic deep-learning-based approach for automated surface inspection. IEEE Trans. Cybern..

[8] Tastimur C., Yetis H., Karaköse M., Akin E. (2016). Rail defect detection and classification with real time image processing technique. Int. J. Comput. Sci. Software Eng..

[9] Jian C., Gao J., Ao Y. (2017). Automatic surface defect detection for mobile phone screen glass based on machine vision. Appl. Soft Comput..

[10] Tsanakas J.A., Chrysostomou D., Botsaris P.N., Gasteratos A. (2015). Fault diagnosis of photovoltaic modules through image processing and Canny edge detection on field thermographic measurements. Int. J. Sustain. Energy.

[11] Mak K.L., Peng P., Yiu K.F.C. (2009). Fabric defect detection using morphological filters. Image Vis. Comput..

[12] Li X., Gao B., Woo W.L., Tian G.Y., Qiu X., Gu L. (2016). Quantitative surface crack evaluation based on eddy current pulsed thermography. IEEE Sens. J..

[13] Yuan X.c., Wu L.s., Peng Q. (2015). An improved Otsu method using the weighted object variance for defect detection. Appl. Surf. Sci..

[14] Win M., Bushroa A., Hassan M., Hilman N., Ide-Ektessabi A. (2015). A contrast adjustment thresholding method for surface defect detection based on mesoscopy. IEEE Trans. Ind. Inf..

[15] Kalaiselvi T., Nagaraja P. (2015). A rapid automatic brain tumor detection method for MRI images using modified minimum error thresholding technique. Int. J. Imaging Syst. Technol..

[16] Bai X., Fang Y., Lin W., Wang L., Ju B.F. (2014). Saliency-based defect detection in industrial images by using phase spectrum. IEEE Trans. Ind. Inf..

[17] Borwankar R., Ludwig R. (2018). An optical surface inspection and automatic classification technique using the rotated wavelet transform. IEEE Trans. Instrum. Meas..

[18] Hu G.H. (2015). Automated defect detection in textured surfaces using optimal elliptical Gabor filters. Optik.

[19] Cen Y.G., Zhao R.Z., Cen L.H., Cui L.H., Miao Z.J., Wei Z. (2015). Defect inspection for TFT-LCD images based on the low-rank matrix reconstruction. Neurocomputing.

[20] Susan S., Sharma M. (2017). Automatic texture defect detection using Gaussian mixture entropy modeling. Neurocomputing.

[21] Song K., Yan Y. (2013). A noise robust method based on completed local binary patterns for hot-rolled steel strip surface defects. Appl. Surf. Sci..

[22] Shumin D., Zhoufeng L., Chunlei L. Adaboost Learning for Fabric Defect Detection Based on Hog and SVM. Proceedings of the 2011 International Conference on Multimedia Technology.

[23] Chondronasios A., Popov I., Jordanov I. (2016). Feature selection for surface defect classification of extruded aluminum profiles. Int. J. Adv. Manuf. Technol..

[24] Gibert X., Patel V.M., Chellappa R. (2016). Deep multitask learning for railway track inspection. IEEE Trans. Intell. Transp. Syst..

[25] Tao X., Xu D., Zhang Z.T., Zhang F., Liu X.L., Zhang D.P. (2017). Weak scratch detection and defect classification methods for a large-aperture optical element. Opt. Commun..

[26] Krizhevsky A., Sutskever I., Hinton G.E. Imagenet Classification with Deep Convolutional Neural Networks. Proceedings of the Advances in Neural Information Processing Systems.

[27] Masci J., Meier U., Ciresan D., Schmidhuber J., Fricout G. Steel Defect Classification with Max-Pooling Convolutional Neural Networks. Proceedings of the 2012 International Joint Conference on Neural Networks (IJCNN).

[28] Faghih-Roohi S., Hajizadeh S., Núñez A., Babuska R., De Schutter B. Deep Convolutional Neural Networks for Detection of Rail Surface Defects. Proceedings of the 2016 International Joint Conference on Neural Networks (IJCNN).

[29] Chen P.H., Ho S.S. Is Overfeat Useful for Image-Based Surface Defect Classification Tasks?. Proceedings of the 2016 IEEE International Conference on Image Processing (ICIP).

[30] Sermanet P., Eigen D., Zhang X., Mathieu M., Fergus R., LeCun Y. (2013). Overfeat: Integrated recognition, localization and detection using convolutional networks. arXiv.

[31] Weimer D., Scholz-Reiter B., Shpitalni M. (2016). Design of deep convolutional neural network architectures for automated feature extraction in industrial inspection. CIRP Ann..

[32] Racki D., Tomazevic D., Skocaj D. A Compact Convolutional Neural Network for Textured Surface Anomaly Detection. Proceedings of the 2018 IEEE Winter Conference on Applications of Computer Vision (WACV).

[33] Lin H., Li B., Wang X., Shu Y., Niu S. (2019). Automated defect inspection of LED chip using deep convolutional neural network. J. Intell. Manuf..

[34] Russakovsky O., Deng J., Su H., Krause J., Satheesh S., Ma S., Huang Z., Karpathy A., Khosla A., Bernstein M. (2015). Imagenet large scale visual recognition challenge. Int. J. Comput. Vis..

[35] Lin T.Y., Maire M., Belongie S., Hays J., Perona P., Ramanan D., Dollár P., Zitnick C.L. Microsoft Coco: Common Objects in Context. Proceedings of the European conference on computer vision.

[36] Erhan D., Szegedy C., Toshev A., Anguelov D. Scalable Object Detection Using Deep Neural Networks. Proceedings of the IEEE Conference on Computer Vision and Pattern Recognition.

[37] Ren S., He K., Girshick R., Sun J. Faster R-CNN: Towards real-time object detection with region proposal networks. Proceedings of the Advances in Neural Information Processing Systems 28 (NIPS).

[38] Redmon J., Farhadi A. YOLO9000: Better, Faster, Stronger. Proceedings of the IEEE Conference on Computer Vision and Pattern Recognition.

[39] Redmon J., Farhadi A. (2018). Yolov3: An incremental improvement. arXiv.

